# Crowdsourcing prior information to improve study design and data analysis

**DOI:** 10.1371/journal.pone.0188246

**Published:** 2017-11-16

**Authors:** Jeffrey S. Chrabaszcz, Joe W. Tidwell, Michael R. Dougherty

**Affiliations:** 1 Department of Social and Decision Sciences, Carnegie Mellon University, Pittsburgh, PA, United States of America; 2 Department of Psychology, University of Maryland, College Park, College Park, MD, United States of America; Emory University Rollins School of Public Health, UNITED STATES

## Abstract

Though Bayesian methods are being used more frequently, many still struggle with the best method for setting priors with novel measures or task environments. We propose a method for setting priors by eliciting continuous probability distributions from naive participants. This allows us to include any relevant information participants have for a given effect. Even when prior means are near-zero, this method provides a principle way to estimate dispersion and produce shrinkage, reducing the occurrence of overestimated effect sizes. We demonstrate this method with a number of published studies and compare the effect of different prior estimation and aggregation methods.

## Introduction

Research activities trade off between accuracy of estimates and cost of information. Limitations on time and funding constrain sample sizes and thereby reduce the information available from any individual study. Additional, extra-experimental information regarding effect sizes would allow researchers to plan appropriate sample sizes and improve Bayesian analyses by constraining and informing prior distributions.

Choosing prior information can be a contentious process. Non-uniform (i.e., informed) prior distributions are often criticized for their subjectivity and may be difficult to estimate in the absence of relevant literature. While empirical Bayesian methods are con tinually improving [1], these methods avoid the near-universal benefit to analytical efficiency gained from including additional prior information. Infinite and uniform prior distributions like those traditionally favored by empirical Bayesians can also be too informative, granting excess credibility to extreme (and sometimes impossible) values of estimated parameters [2]. For sparse data or small sample sizes, uniform prior distributions can produce inappropriate parameter estimates.

Even when using frequentist analysis or uninformed priors in Bayesian analysis, additional information about the study can improve sample size planning. Many hypothesis-driven studies focus on establishing that a particular parameter does (or does not) differ reliably from zero. To use an example from the current research, perhaps desire for an object reduces the perceived distance to that object [3]. If desire influences perception, a thirsty person should claim that a bottle of water a fixed distance from them is closer than a less thirsty person does, on average. To test the theory that desire alters estimates of distance, a researcher does not have to accurately predict participants’ estimated distance to a water bottle, he or she only has to provide evidence that the perceived distance to a water bottle is reliably less on average for thirsty people than for their less-thirsty reference group. By randomly assigning individuals to thirst or satiety, the researcher can ignore other factors that influence perceived distance (e.g., spatial reasoning, the actual distance, etc.) and build a model that predicts estimated distance with only an indicator as to the thirst of a given individual.

Ideally, a researcher would look to previous research to estimate an expected effect size, both to use in a Bayesian analysis and to plan for a sample of sufficient size to reliably estimate the hypothesized non-zero effect. Uncontested prior information is not always available: A novel outcome measure or widespread disagreement within the literature would both make the choice of prior (or expected effect size) contentious. Rather than planning studies based on guesses about the range of observable effect sizes—or worse, hoping for optimistically-large effect sizes—researchers would benefit from an inexpensive and objective method for obtaining impartial estimates of effect size. Better planning would reduce the resources spent investigating large effects, allowing more resources to be allocated to accurately estimate small or highly variable effects or saved when the existing resources would be insufficient to gather the necessary information relevant to a parameter of interest.

Estimating reasonable priors is a forecasting problem and amenable to crowdsourcing or other group aggregation strategies. While no individual is likely to produce well-calibrated parameter estimates in the way that a scientific study would, individuals do have substantial experience making predictions about an uncertain world. Many studies demonstrate that combining individual subjective probability estimates can produce improved forecasts [4]. While the unweighted linear average of individual forecasts is one successful strategy [5], more recent research has demonstrated the effectiveness of multilevel models at simultaneously recalibrating individual subjective probability estimates and generating an aggregated forecast [6]. This is, at least in part, because multilevel structure allows for efficient use of information when we have little information about individual forecasts compared with the group average [7]. Rather than relying on completely uninformed prior distributions or guesses about effects sizes, easily-collected individual estimates could be aggregated and used to both plan studies and increase the information included in data analysis.

The current article discusses two ways that prior distributions on effect sizes can be elicited and used to improve psychological studies that are tested by comparing two groups on a continuous outcome. Though testing and discussion are limited to comparisons between two groups, this method could be scaled to any number of analyses. We elicit effect sizes from participants using abstract and concrete prompts and explore aggregation of these estimated quantities using both maximum likelihood estimation and hierarchical Bayesian modeling. These methods could be widely implemented with existing technology for minimal cost.

## Materials and methods

### Participants

We recruited 48 undergraduates from the Psychology Department participant pool at the University of Maryland, College Park. Participants received partial course credit for taking part in the study. The University of Maryland Institutional Review Board approved all study procedures under Protocol Number 09-0493.

### Procedure

Participants who signed up for the study received a link to a Qualtrics survey. This link guided participants through a short training on our elicitation method, followed by a series of questions that asked participants to give estimates of effect sizes and then corresponding probability estimates of the elicited quantities. Following successful completion of the study, participants saw a “thank you” message and received course credit.

### Elicitation

For each experimental question, participants saw a total of three separate prompts:

1. An abstract prompt that posed the central, theoretical question of a given study;
2. A concrete prompt that explained the relevant control-condition context and introduced the outcome and measurement scale; and,
3. A concrete prompt that explained the relevant treatment-condition context and repeated the outcome and measurement scale.

In response to the abstract prompt, participants produced a single response on the 0–100 scale using a slider, with 0 anchored at, “this statement is definitely not true,” and 100 anchored at, “this statement is definitely true.” We used the following abstract prompts:

1. The font of a survey about negative personality traits alters the number of negative personality traits that respondents admit to having [8].
2. Desire for an object changes the perceived distance to that object [3].
3. Thinking about luxury material goods increases depressive feelings [9].
4. Superstitious belief increases golf performance [10].
5. Infants can map increasing line lengths to a mental number line [11].
6. Additional competitors reduce individual motivation to compete [12].
7. Verb tense used to describe past actions can influence memory for those past actions [13].
8. Physically enclosing an upsetting object reduces negative emotions associated with that object [14].

Each prompt corresponds to studies in an article included in a recent examination of articles in the journal *Psychological Science* [15]. These questions all presumably merit investigation by virtue of their appearance in a high-impact journal. The corresponding concrete prompts each included two elicitation steps. For each condition of each prompt, participants estimated three outcome measurements: Their best guesses for the outcome in a particular condition, their minimum guesses, and their maximum guesses. We required that these responses be weakly monotonic, such that the best guess could not be smaller in magnitude than the minimum or larger in magnitude than the maximum. After giving these estimates, participants gave cumulative probability estimates corresponding to three new quantities that we based on the initial elicited values. These new values were defined as $c_{1}=L+\frac{H-L}{6},c_{2}=\frac{H-L}{2},c_{3}=H-\frac{H-L}{6}$, where H is the maximum elicited value for a given estimate and L is the minimum. Again, we required that the probability estimates be weakly monotonic, such that *p*(*c*_1_) ≤ *p*(*c*_2_) ≤ *p*(*c*_3_). We used the following concrete prompts, with the alternative text that defines the manipulation for each prompt appearing in parentheses:

1. Out of 30 possible traits, how many negative traits would the average person admit to having (while reading the traits in unclear text)?
2. How far away would the average person (who had just eaten pretzels) estimate a bottle of water that is truly three feet away?
3. On a range of 0 to 20, how depressed is the average person (after viewing pictures of luxury good)?
4. Out of 10 attempts, how many putts at a distance of 3 feet would the average person sink (after hearing that they are using a lucky ball)?
5. How long would infants look at a series of five pictures in which the number of shapes decreases/(increases) in each successive picture?
6. How long on average will an individual in a group of 10/(100) people take to complete a timed, 10-question math quiz?
7. One a scale of 1 to 10, where higher numbers indicate higher hostility, how hostile would an average person rate a Black man’s actions after being asked what they did/(were doing) in a past interaction with a Black man?
8. On a scale of 1 to 5, where higher numbers mean more negative feelings, how negatively would an average person feel after reading a news article about the death of a child (and then enclosing that article in an envelope)?

### Aggregation

We modeled elicited probabilities in two ways. The first used maximum likelihood estimation to find the best point estimates for each participant’s mean and standard deviation parameters independently for each condition. To derive maximum likelihood estimates, we assumed Gaussian forms for participant prior distributions and calculated *μ* and *σ* for each participant, question and condition by minimizing squared error loss for the differences between elicited probabilities and calculated probabilities corresponding to a given quantile prompt (i.e., *c*_1_, *c*_2_, and *c*_3_) from the generated *μ* and *σ*. We then aggregated the participant parameters by taking the independent medians of the *μ* and *σ* parameters, producing a single, normally-distributed prior for each condition in each experiment [16, 17]. Though the assumption of Gaussian beliefs is quite strong, we avoid any interpretation of a “best guess” by using more information from the elicitation method, allowing us to weight participants by their confidences in the consensus distribution.

**Fig 1:**
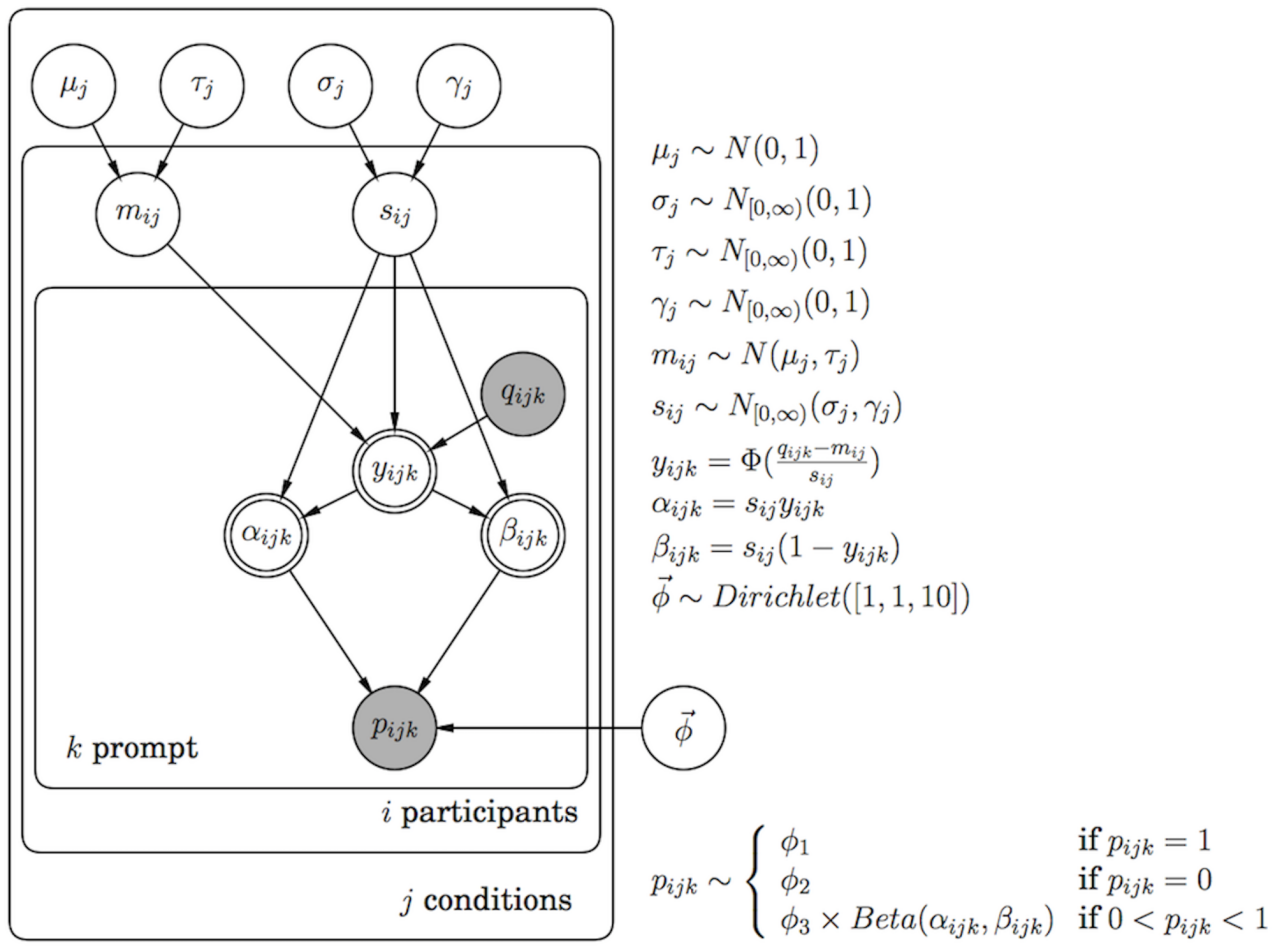
Graphical model [21] of the hierarchical Bayesian model applied to each question independently.

We estimated (and simultaneously aggregated) participant-varying parameters using a hierarchical Bayesian model (HBM, Fig 1). Code and data for these models are available at https://github.com/jchrszcz/papers/tree/master/chrabaszcz_csp. We fit these models using Stan [18, 19] and performed all analysis with R [20]. In these models, we defined the likelihood as a mixture of a Beta distribution with parameters that depend on the *c*_1_, *c*_2_, and *c*_3_ prompts and corresponding probability estimates (*q*_*ij*_ and *p*_*ij*_, respectively, in Fig 1) that each participant saw and a Bernoulli distribution that did not depend on these prompts. The Bernoulli mixture is included to remove exact zero and one values from elicited probabilities, which are incompatible with the study design. We include a mixture parameter, $\vec{\phi }$, to separately model the probability of producing responses of 0, 1 or a valid response from the estimated Beta distribution. The Dirichlet prior on *ϕ* corresponds our expectation that fewer responses will be exactly 0 or 1. Participants respond to quantile values that are greater than the elicited minimum and less than the elicited maximum for each question, so providing probability estimates that are incompatible with more extreme quantile values should never occur. Elicited probabilities (with the contaminant zeros and ones accounted for) are predicted using Φ^−1^-transformed combinations of the participant-varying quantile prompts: the *m* and *s* parameters. While prompts were fixed as part of the elicitation process, we modeled the *m* and *s* parameters as samples from normal distributions with centers *μ* and *σ* and standard deviations *τ* and *γ*, the estimated variances on participant-wise parameters. This allowed us to use the *μ* and *σ* hyperparameters as the consensus distribution for each condition and question. For all following calculations, we use the median of these hypermaters to reflect the parameter value.

We fit a separate model for each question. The only parameters consistent across all participants and both conditions for each question are *ϕ* and *ρ*, which measure the probability of responding exactly zero or one. We elicit probabilities corresponding to finite effect size estimates which are assumed to be normally distributed with a mean and standard deviation that vary by participant and condition for a given question, so responses of exactly zero or exactly one should never be observed. The model we used is a Bayesian version of an inflated regression model, which are used to model data assumed to arise from a mixture of two error distributions. In this case, we assume that participant probability estimates will have residuals that result either from a Bernoulli distribution (which accounts for the zero and one responses) or a Beta distribution (which applies for all other response values).

We scaled all quantile prompts (*c*_1_, *c*_2_, and *c*_3_) for each question separately prior to fitting the model, then reversed the transformation to generate unstandardized estimates for each question. This scaling allowed us to speed convergence by assuming a normal prior distribution with zero mean and one standard deviation independently for each condition mean and a half-normal prior distribution with zero mean and one standard deviation for eacg condition standard deviation [22]. It also enabled us to use consistent prior distributions across the differently-scaled questions under consideration.

## Results

**Fig 2:**
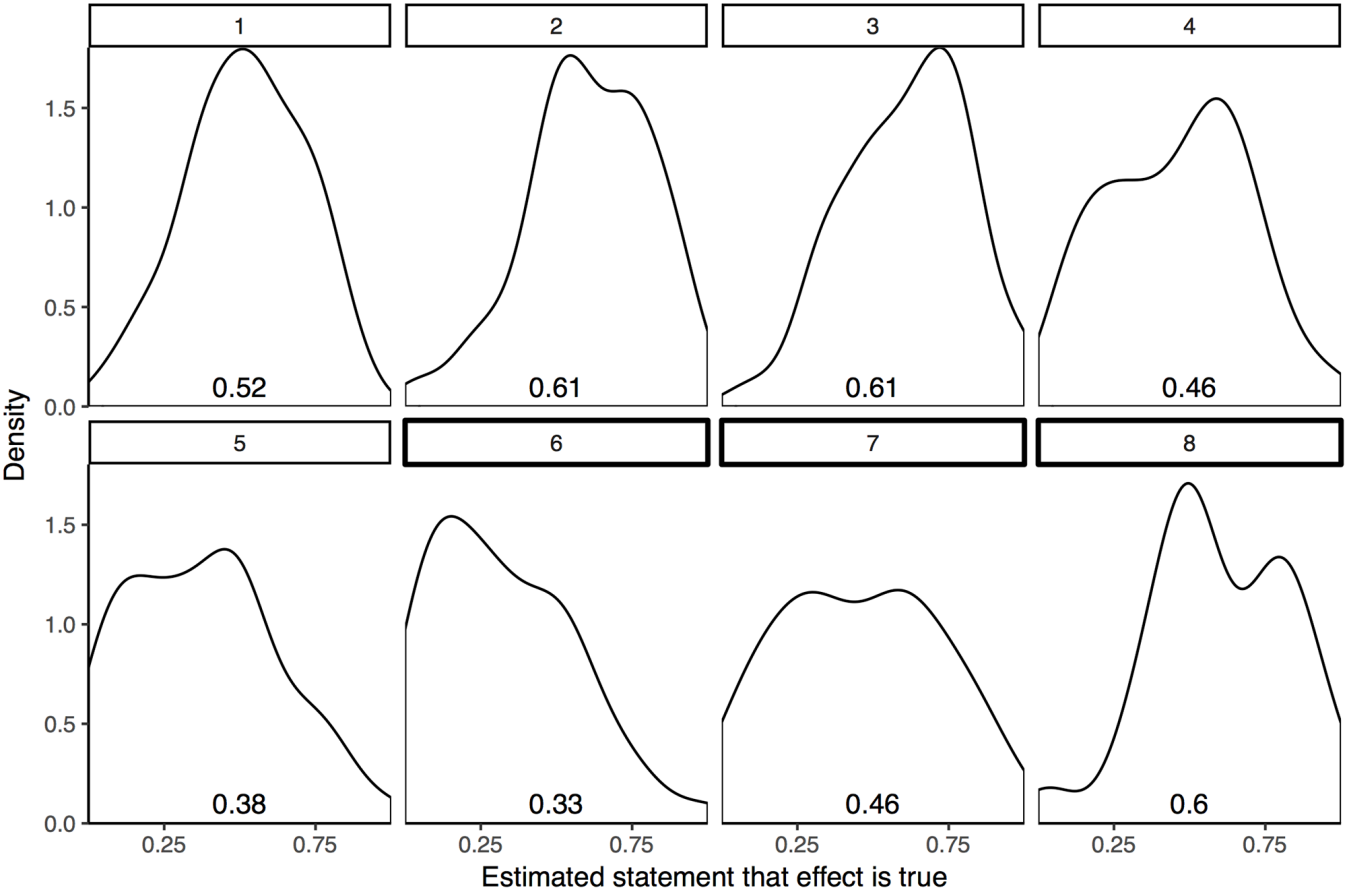
Densities of participant responses to abstract prompt. Superimposed quantities give the mean probability for the estimated truth of each statement.

Abstract elicitation produced a range of belief centered approximately at chance for most questions. Participants were slightly more credulous than average for questions 2, 3, and 8 and more skeptical on questions 5 and 6 (Fig 2). These simple averages provide a calibration check for the more complicated models. If participants held strongly divergent beliefs about the studies as a function of the elicitation method, then we would either doubt the methods and modeling or require an explanation for the discrepancy. Though we believe the models used to form consensus distributions for each question are giving more accurate estimates than the raw responses to the abstract prompt, both methods suggest that participants have a range of prior belief and that, on average, people believe that the experimental effects will be either very small or highly variable.

**Fig 3:**
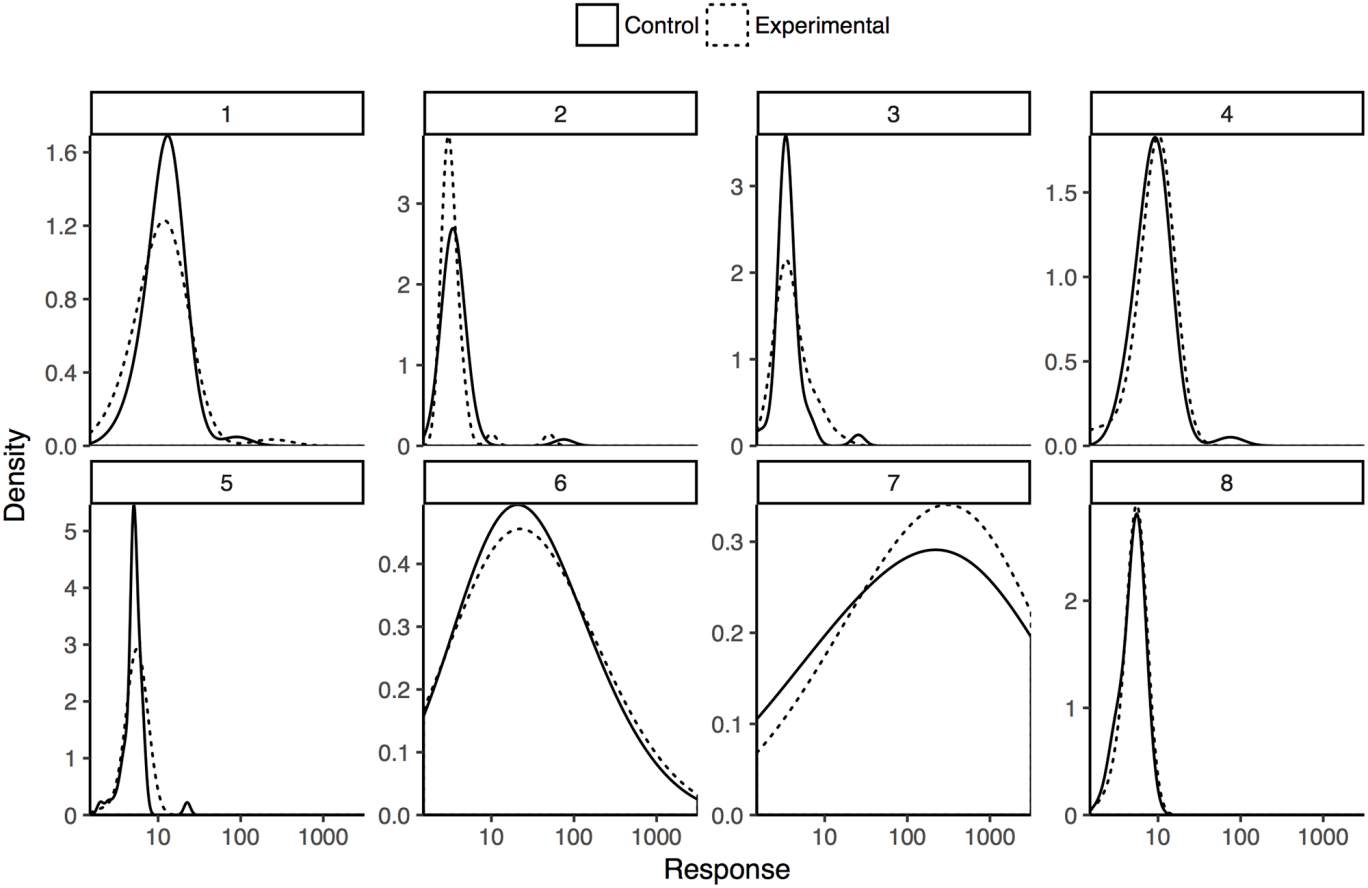
Densities of raw best guess responses to concrete elicitation, colored by condition and faceted by study question. The horizontal axis is logarithmically scaled to minimize the visual effect of extreme estimates.

We analyzed raw participant responses to concrete prompts in three different ways. One initial approach is to examine the graphical distributions of “best guesses” from the first elicitation step (Fig 3). This method has at least two problems. We have no way of comparing confidence in these estimates. Presumably some participants are more certain of their estimates than others, but we have no way of knowing the relative confidence in these point estimates. We also have no way of knowing what a “best guess” reflects. If participants have a symmetrically-distributed belief regarding the elicited values, then the best guess will represent both the mean and median of the distributions. This is not necessarily the case, however; participants could implicitly use an asymmetric cost function or have skewed distributions of belief regarding the elicited parameters. Aggregating participant belief into a prior distribution on an effect size is intractable without further information in any of these scenarios. Despite these problems, Fig 3 is consistent with responses to the abstract prompt. The densities correspond to each condition show substantial overlap and, in most cases, high variability. These graphs provide a second indication that prior estimates of effect size will be more variable than large.

**Fig 4:**
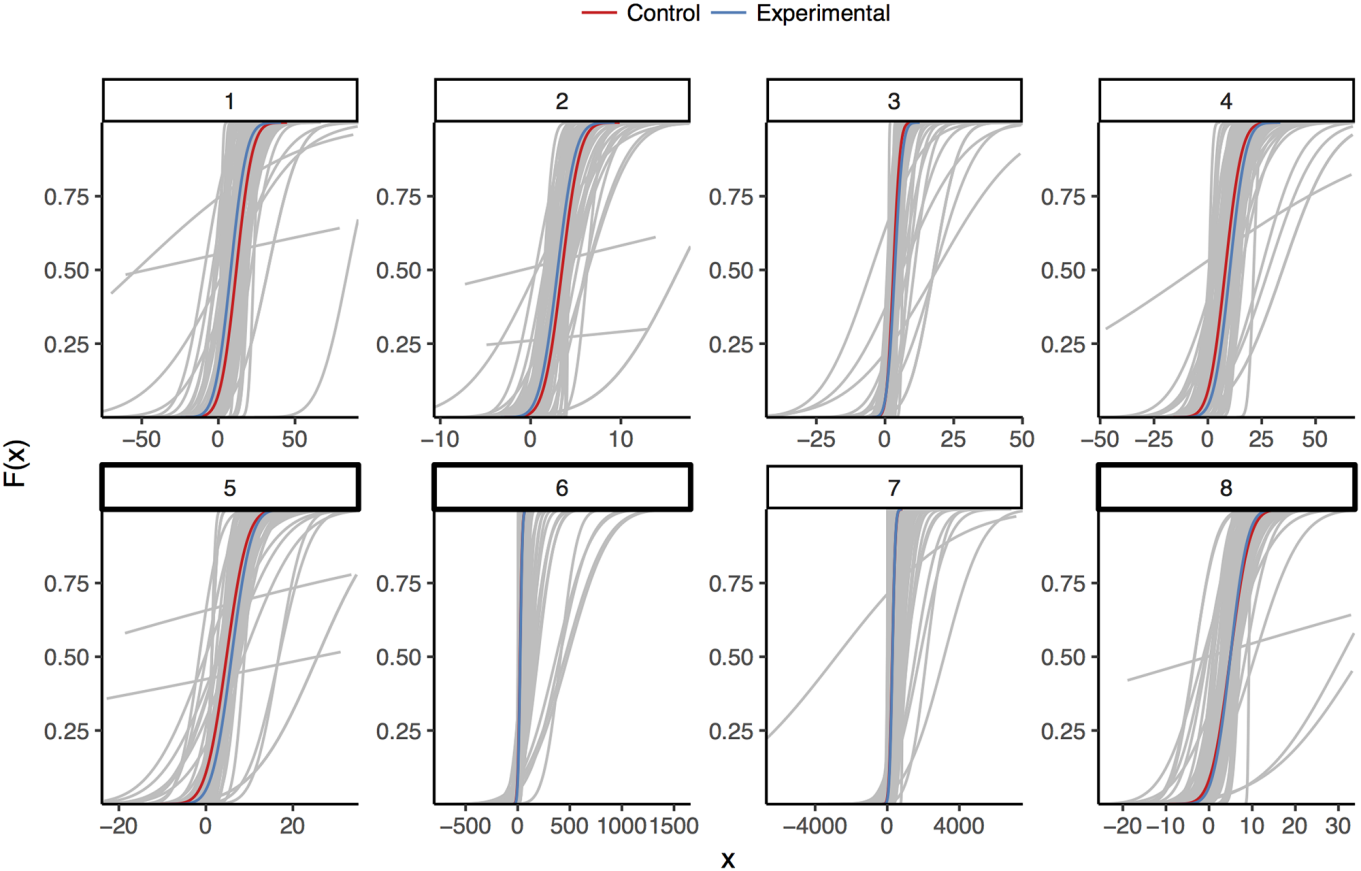
Cumulative normal curves generated from maximum likelihood estimates for each participant and condition, faceted by question. Participant curves for both conditions are in gray, while the curves corresponding the median mean and standard parameters in each condition are used to create consensus curves, colored by condition.

Another way to look at participant responses is to model them using maximum likelihood estimation (MLE). We can see in the cumulative normal curves for each participant and the associated median aggregation curves for each condition that participants hold a range of prior beliefs regarding the effects of these manipulations (Fig 4). MLE produces consensus distributions that vary widely between questions. Some of the participant curves demonstrate rather extreme values for the modeled parameters. A drawback of MLE is that each participant is modeled independently of the others. This particular model carries with it the assumption that we learn nothing about other participants from a given participant’s responses [7, 23], despite the fact that we have quite a bit of information about the participant averages relative to the information from a given individual.

**Fig 5:**
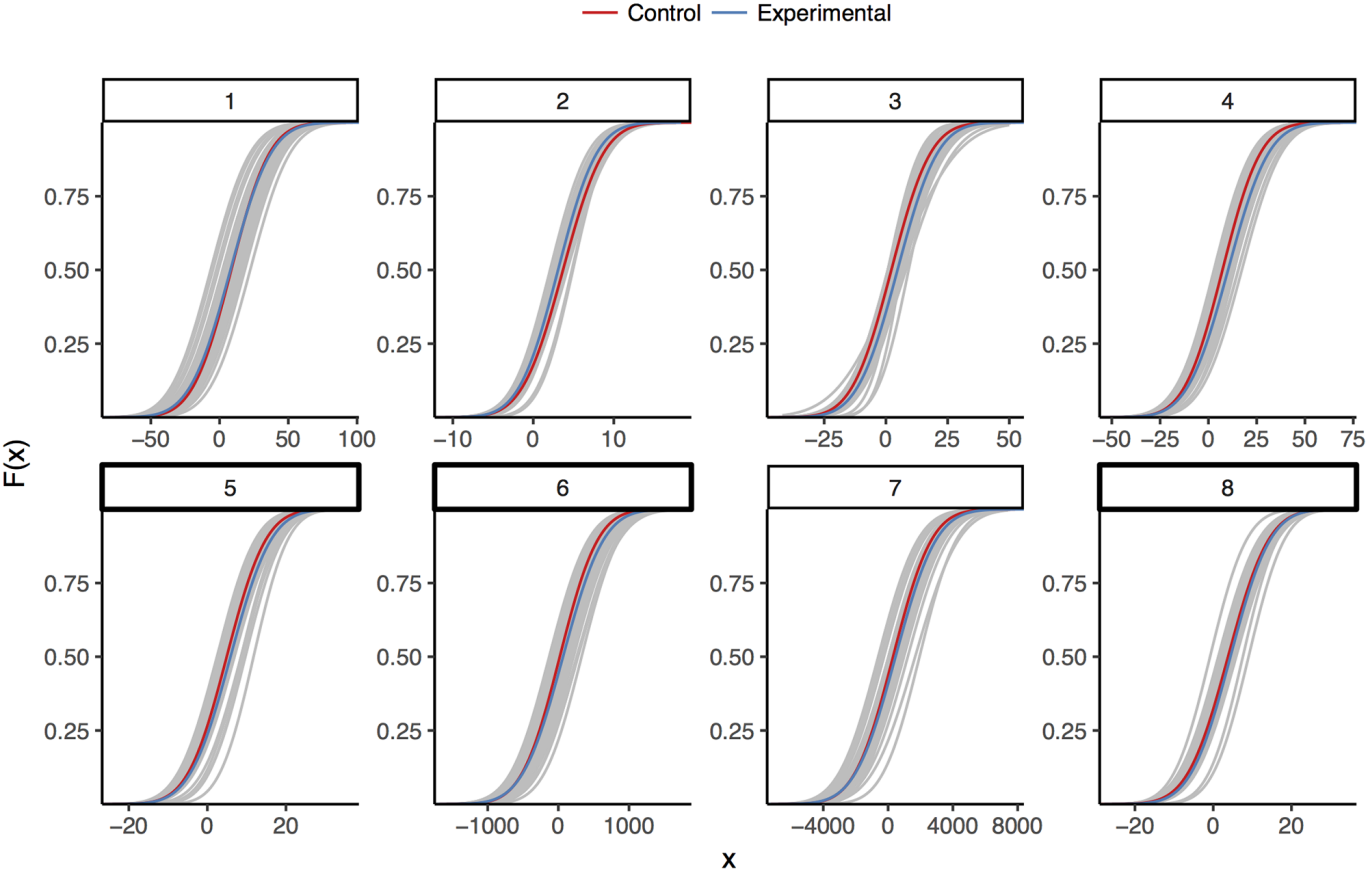
Cumulative normal curves generated from a Bayesian multilevel model fit independently to each question. Participant curves for both conditions are in gray, while the curves corresponding the median mean and standard parameters in each condition are used to create consensus curves, colored by condition.

**Fig 6:**
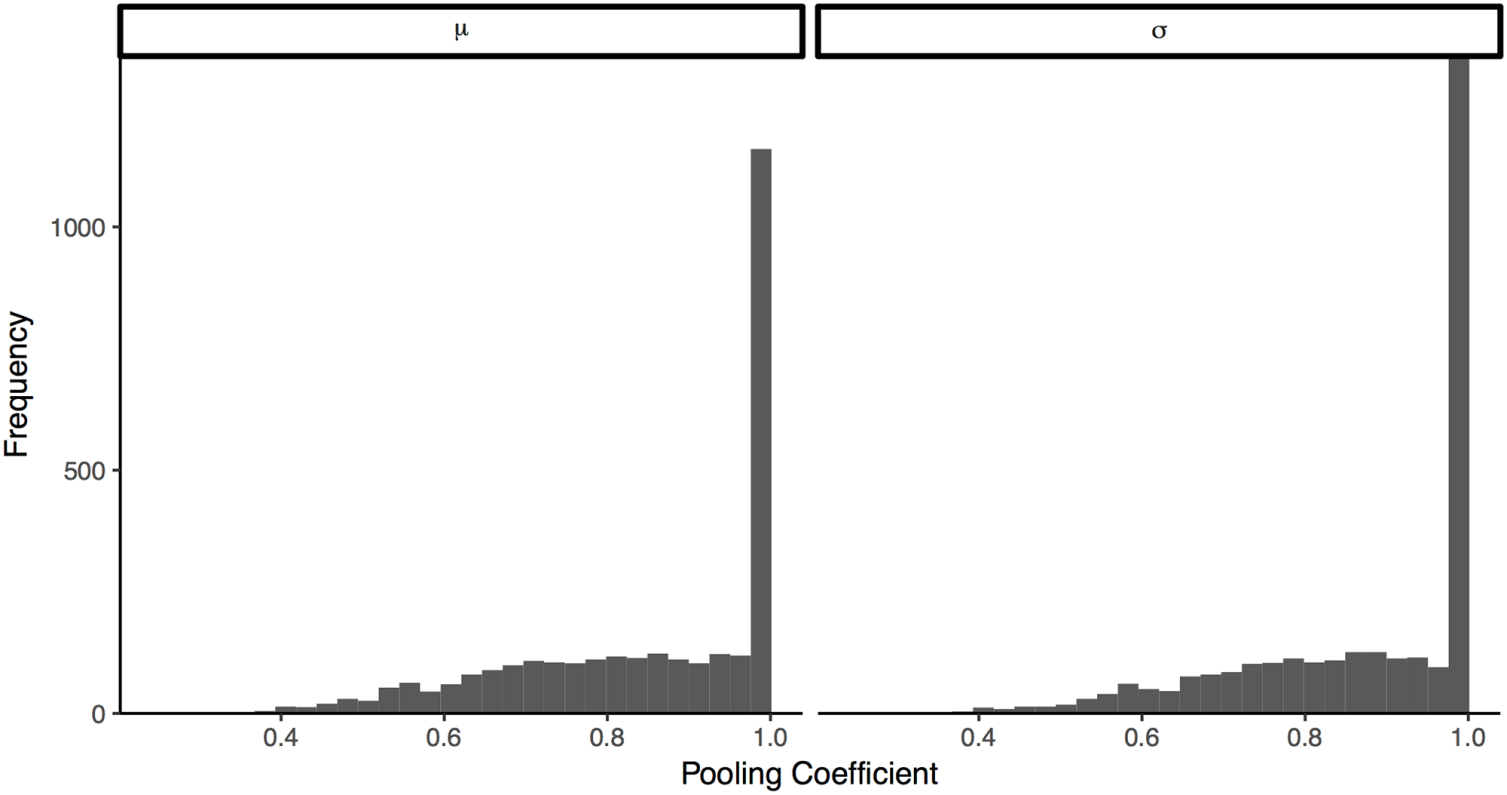
Example pooling coefficients for m and s parameters in the control condition of question 4. The pooling coefficient is a measure of how much a given parameter is shrunk toward the grand means, in this case *μ* and *σ*: 0 is no pooling and 1 is infinite pooling.

By assuming estimated means and standard deviations for each participant are drawn from shared distributions in the HBM, we can partially pool our estimates to both produce more realistic individual parameter estimates and aggregate them based on the shared distribution parameters. Fig 5 shows the cumulative normal curves for participants and the implied consensus distributions using HBM with partially-pooled multilevel structure. Relative to MLE, Bayesian participant estimates are smoothed toward the grand mean of each parameter (Fig 6). The hyperparameter consensus distributions from the Bayesian model are reasonably close to those from median aggregation with MLE (Table 1). These estimated effect sizes are reasonably consistent with the mean abstract elicitation probabilities, which provides some minimal assurance that participants are consistent across elicitation methods and that the aggregation procedure is not pathologically mis-calibrated.

**Table 1:** Difference in conditional means, pooled standard deviation, and effect sizes as estimated by the published study and aggregated participant forecasts using MLE and HBM. Standation deviation (SD), Effect size (ES).

	Empirical			MLE			HBM		
Q	Mean	SD	ES	Mean	SD	ES	Mean	SD	ES
1	0.01	0.28	0.05	-3.61	11.63	-0.31	-0.70	32.39	-0.02
2	-2.90	9.58	-0.30	0.47	2.97	0.16	2.54	17.90	0.14
3	0.36	0.90	0.40	2.11	8.41	0.25	2.96	22.78	0.13
4	1.85	2.86	0.65	1.17	5.21	0.22	0.93	11.32	0.08
5	3.80	10.86	0.35	1.86	24.55	0.08	45.83	627.11	0.07
6	4.20	12.66	0.33	6.89	192.04	0.04	173.62	2696.71	0.06
7	1.11	8.31	0.13	0.01	4.76	0.00	0.60	11.43	0.05
8	-0.46	1.29	-0.36	-0.48	2.24	-0.22	-0.60	5.57	-0.11

Table 1 shows the difference in conditional means, pooled standard deviations, and effect sizes for each question based on the data and two aggregated estimates. Both MLE and HBM produces mean and standard deviation estimates that are within an order of magnitude of the empirical estimates in most cases. Both aggregation models produce smaller average estimated effect sizes compared with the empirical observations, a product of proportionally larger estimated standard deviations.

**Fig 7:**
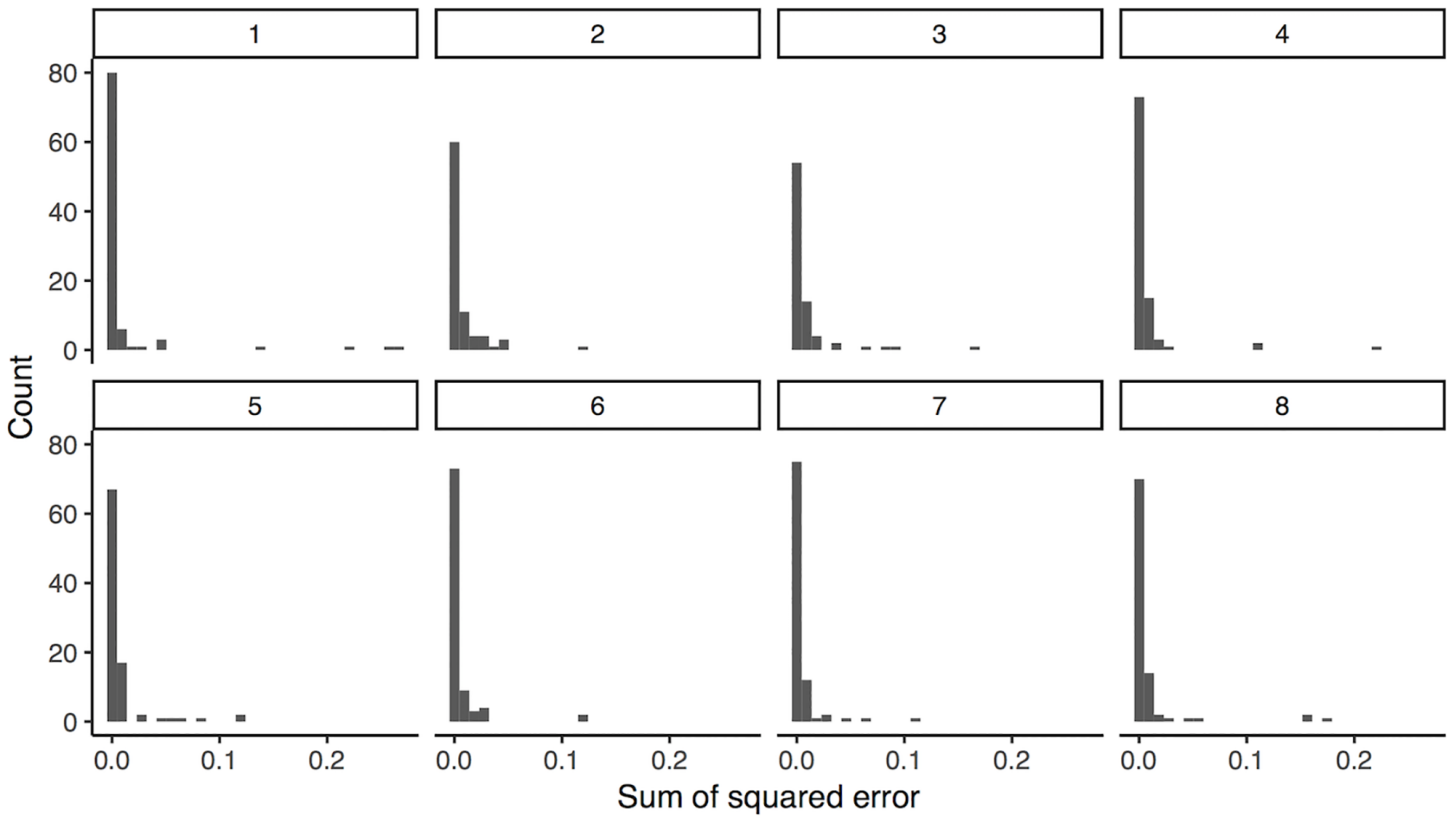
Sums of squared error by participant, collapsing across conditions, for each of the eight questions.

**Fig 8:**
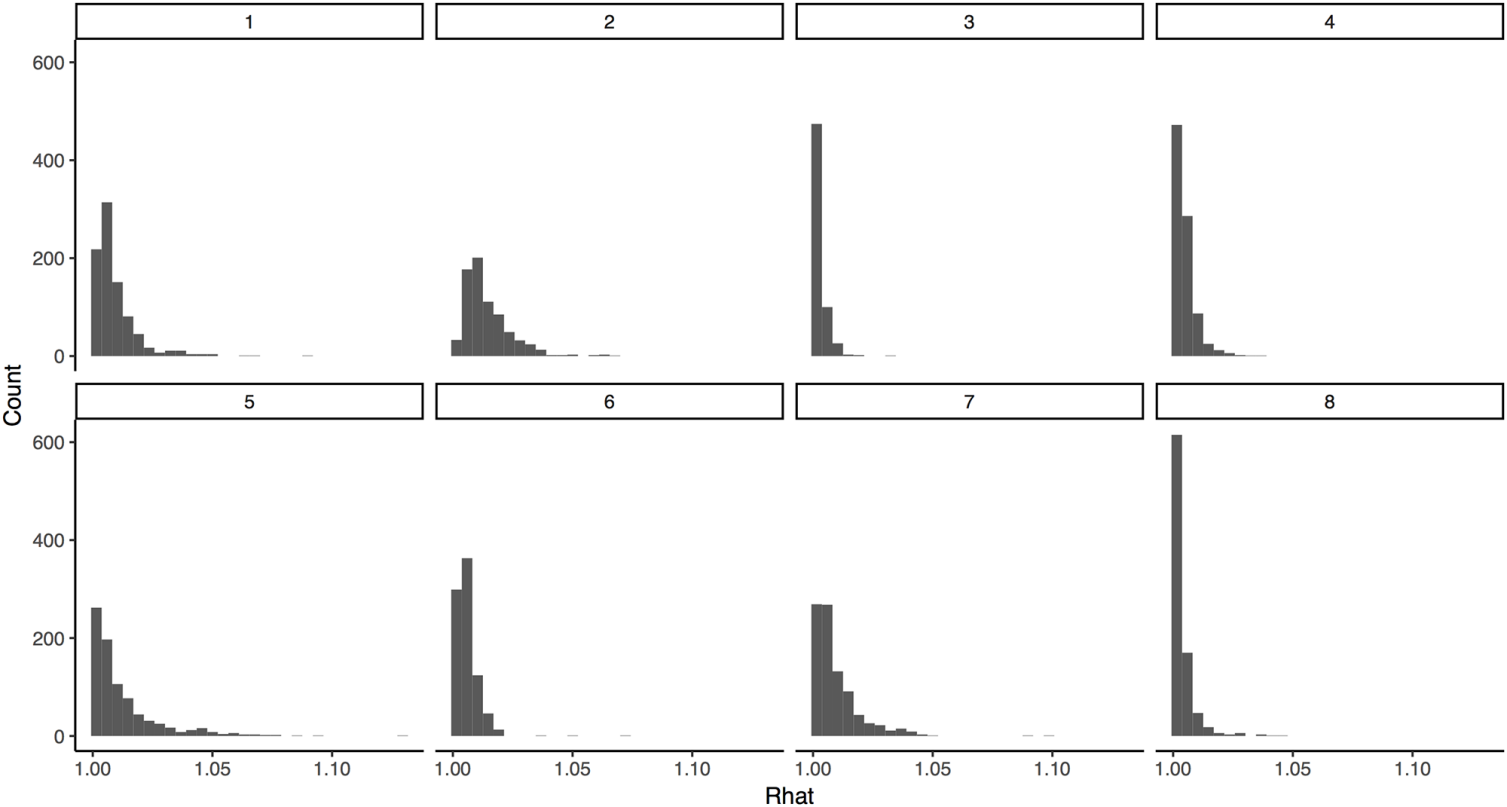
Distributions of $\hat{R}$ for each model.

Both the maximum likelihood and hierarchical Bayesian methods of modeling the data showed reasonable correspondence to the data. The maximum likelihood model was able to explain greater than 99% of the variance in roughly 97% of elicited distributions. Fig 7 shows the distribution of the sums of squared error for each question. All Stan models had $\hat{R}$ of less than 1.1 and effective *n* of more than a few hundred based on models of five independent chains each, indicating sufficient mixing between chains and sufficient independent samples of marginal distributions for each parameter to support inference (Fig 8 for $\hat{R}$ distributions by model). Each chain was initialized with pseudo-random draws from the priors based on CPU time.

**Fig 9:**
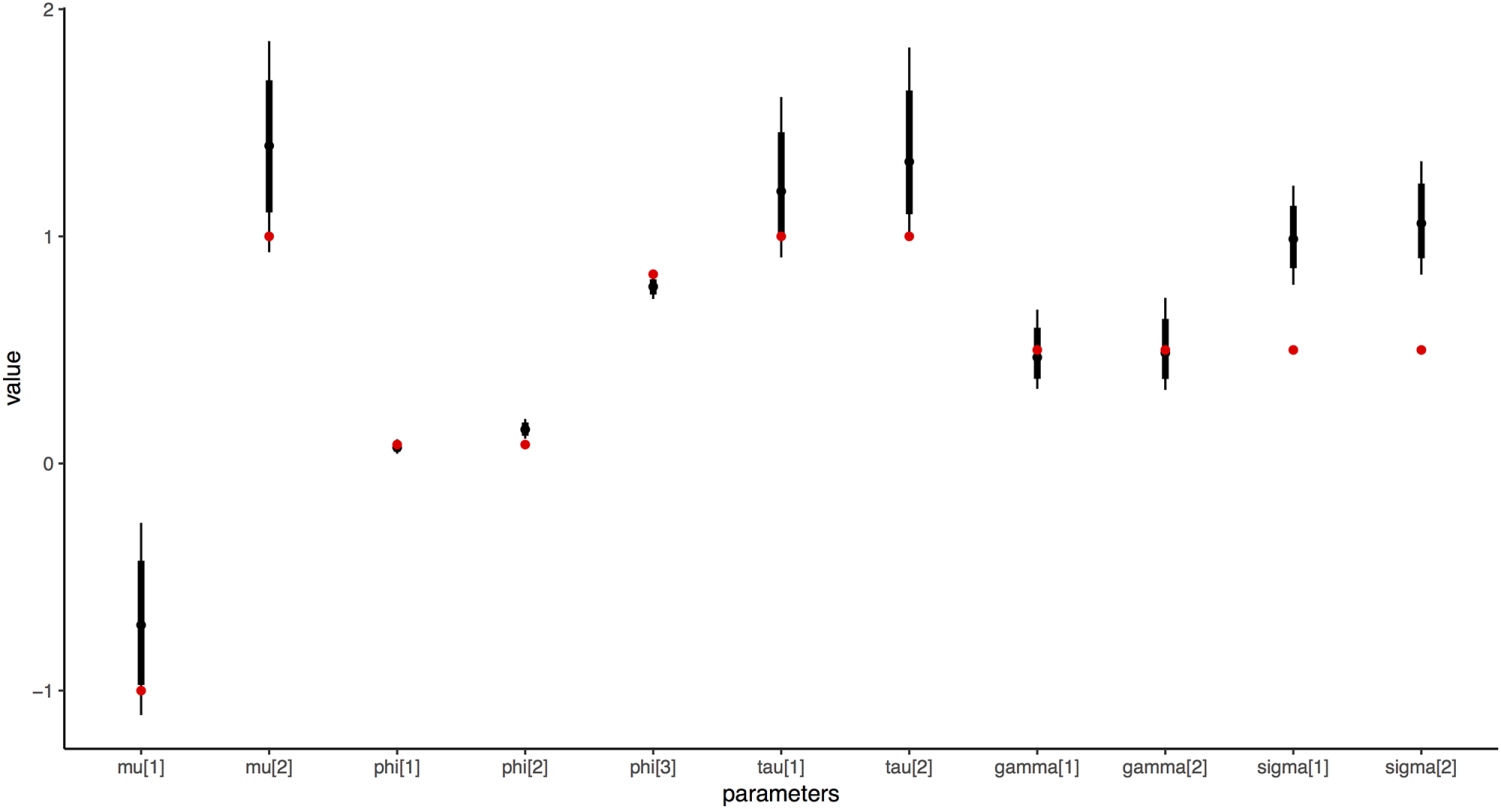
True parameter values (red) for each parameter compared with the HBM posterior estimates for the four top-level hyperparameters fit to one set of twenty subjects simulated from fixed parameters.

The HBM produced aggregated parameter that are visually similar to the average abstract prompt responses, average “best guess” responses, and median-aggregated MLE estimates. We also produced a very simple simulation to show that the HBM can recover known parameters. Fig 9 compares the posterior distribution of the HBM to to the true parameter values (red points) for each of the top level parameters. In this figure, the model is fit to three responses for two conditions for each of twenty simulated subjects whose responses are generated using the priors listed in Fig 1. With the exception of *σ*, which is biased too close to zero a half-normal prior given the small simulated sample size, we find reasonable correspondence between the true and estimated parameters.

Though our focus is not on the specific studies but on the general process of planning and interpreting experimental studies, we examined the power of these eight studies with the existing samples sizes and assuming true effect sizes based on the observed effect size or the priors elicited and aggregated with MLE or HBM. Table 2 gives these quantities. Empirical estimates of effect size yield low power in half of the observed samples. The empirical effect sizes are based on relatively small samples, though, which yield biased effect size estimates [24]. If we base our power calculations on the effect sizes from our survey responses and ignore the study information, the power for these studies is even lower. Our elicited and aggregated effect sizes suggest the need for much larger samples than do the empirical effect size estimates.

**Table 2:** Power (Pwr), probability of sign error (Sn), and proportion of magnitude error (Mag) for each of the eight studies.

	Empirical			MLE			HBM		
Question	Pwr	Sn	Mag	Pwr	Sn	Mag	Pwr	Sn	Mag
1	0.057	0.242	9.236	0.4	0.001	1.559	0.051	0.372	20.082
2	0.811	3 × 10^−6^	1.114	0.318	0.001	1.752	0.263	0.002	1.924
3	0.789	9 × 10^−6^	1.135	0.409	0.001	1.539	0.142	0.018	2.766
4	0.909	5 × 10^−6^	1.057	0.197	0.008	2.23	0.068	0.143	5.787
5	0.362	0.001	1.639	0.063	0.183	6.923	0.062	0.191	7.064
6	0.804	4 × 10^−6^	1.124	0.06	0.201	7.817	0.084	0.078	4.416
7	0.162	0.012	2.556	0.05	0.482	148.826	0.066	0.151	6.289
8	0.884	9 × 10^−7^	1.069	0.475	2 × 10^−4^	1.438	0.156	0.013	2.604

Table 2 also gives the probability of mis-estimating the sign of each effect (Type S errors) and the magnitude of overestimation (Type M errors) for each study based on retrospective power estimates and prior effect sizes [25]. Across estimation methods, most of the studies under consideration have low chances for sign errors. The effect sizes are likely to be overestimated, however; while the Type M error magnitude is modest based on empirical estimates, HBM aggregation suggests that most effects are estimated at a minimum of nearly double the true value.

Elicited effect sizes could be used to plan sample sizes for future studies. Table 3 shows the sample sizes included in each study and those required to reach the recommended 80% power using the effect sizes estimated from the studies and our aggregation methods [26]. These sample sizes reflect the effect sizes in Table 2. Sample sizes would generally need to be larger, and sometimes much larger, to achieve the recommended power based on the empirical estimates (which are likely optimistic). MLE- and HBM-aggregated priors suggest even larger samples. HBM estimates are less extreme than those from MLE; while the lower recommendations from HBM are not as low as those from MLE, HBM also limits recommendations to thousands rather than the tens of thousands of participants that MLE implies would be required for sufficient power in experiment seven.

**Table 3:** Sample size required to reach 80 percent power for based on the empirical and consensus-modeled effect sizes.

Q	N	Empirical	MLE	HBM
1	33	3,517	81	16,951
2	90	86	309	388
3	50	49	124	466
4	28	19	157	1,165
5	24	64	1,372	1,468
6	74	71	6,090	1,891
7	56	439	1,655,985	2,866
8	80	61	169	673

For samples as small as those observed, the elicited effect sizes can have substantial influence on test statistics. Table 4 presents the *t*-values estimated in each study, compared with those estimated including the prior information from our elicitation and aggregation methods. The MLE and HBM esimated t-values are calculated by calculating an unequal-variances t value using a weighted average between empirical means and standard deviations and those estimated using MLE and HBM. The empirical values are given the full weight of the reported sample size, while the MLE and HBM estimates are weighted as a single observation. In all of these examples, a *t*-value with a magnitude of 2.00 (and as low as 1.96) would be significant at the *p* = .05 level, which is the traditional decision point in psychological sciences. In our small sample, most studies no longer feature a significant difference between the means of the control and experimental groups. That is not to say there is no difference in means; simply that these studies are not informative about the differences when prior information is taken into account.

**Table 4:** t-values as reported by the original studies and as recalculated using our elicitation and aggregation methods.

Q	Empirical	MLE	HBE
1	2.26	-0.21	-0.01
2	-2.00	-2.78	-2.73
3	2.00	1.46	0.80
4	2.14	2.86	2.50
5	2.37	1.56	0.21
6	2.02	2.76	0.18
7	8.00	0.95	0.96
8	-2.24	-2.10	-2.05

## Discussion

Our goal is to assess a method of gathering inexpensive effect size estimates for use in psychological research. The current aggregation method via HBM yields a wide range of prior belief as expressed by our participants that is consistent with, but likely more accurate than, simpler aggregation methods. This procedure is useful and potentially applicable in all cases about which participants can reasonably be informed.

We elicit sufficient information from participants to model their beliefs as a Gaussian function. This potentially allows for a variety of different aggregation methods. We have focused on hierarchical Bayesian modeling for a few reasons. HBM can model both the participant-wise parameters and aggregate them simultaneously. Partial pooling for information between participants’ parameters represents a reasonable default aggregation method for experimental studies, though other methods that make explicit use of decision analysis may be more appropriate in some circumstances. HBM with boundary-avoiding priors also produces consensus estimates that are quite similar to median aggregation using MLE. This convergence is reassuring, though HBM also gives much more sensible participant-wise parameters estimates in cases where those are of interest. Individual participant parameter estimates are sometimes quite extreme using MLE, owing in part to the relatively little data used to fit each individual’s parameters. These extreme estimates are the justification for using the median instead of the mean to aggregate in MLE; they are also justification for preferring partial pooling in HBM, which produces fewer extreme estimates. This is important for accurately estimating the variability of effect size belief, which in turn influences how informed the estimated effect size will be in subsequent analysis and how large of a sample would be required to reliably detect an observed effect size. Though aggregated estimates for MLE and HBM were very similar, some substantial differences exist in participant-wise parameters. In cases where individual differences matter, such as when investigating expertise, robustness, consensus on inferences, or other phenomena that involve estimation of individual predictions, HBM should produce more calibrated estimates.

This elicitation method is not limited to comparisons between two groups. A similar technique could be used to generate priors for nearly any linear model. The only limitations are practical: It is much more difficult to elicit participant beliefs when they must be conditioned on other elicited beliefs, as they would have to be to correspond to a model with multiple independent predictors.

The methods we have discussed are not without limitations. The most important modeling assumption we make is the Gaussian form of participant beliefs. Participants might not even have a distribution of belief, let alone beliefs that take on the distribution we have assumed. Gaussian distribution of belief is a convenient assumption that should be more thoroughly validated. Other methods with different assumptions like kernel density estimation or Gaussian process modeling could be used to generate participant estimates. These techniques generally require more data to get reliable fits than the model under discussion, introducing different limitations. We also assume that our participants are able to make informed predictions about the studies of interest. Participants live in the world and have some experience with scenarios like those we describe, so this does not seem unreasonable. This method of generating priors would not be applicable in scenarios where participants would not have any relevant experience. We further assume the prompts used in elicitation do not substantially bias reported beliefs. This could be mitigated by using separate forms for elicitation, though more complicated elicitation is counter to the goal of gathering inexpensive prior information to improve analysis of experimental data.

## Conclusion

Psychological research would benefit from unbiased estimates of effect sizes prior to gathering expensive, experimental data. This information would improve study planning by reducing the tendency toward optimism in estimating effect sizes when calculating the necessary sample size to detect reliable effects. The same information can be incorporated into Bayesian analyses, providing maximally-informed parameter estimates without potentially biasing the analysis by using optimistic priors while improving efficiency relative to empirical Bayesian analysis.
